# Supplementary material for: Integrating depth-dependent protist dynamics and microbial interactions in spring succession of a freshwater reservoir
Source: Environ Microbiome. 2024 May 8;19:31. doi: 10.1186/s40793-024-00574-5 (PMC11080224; doi:10.1186/s40793-024-00574-5)
Supplement: Supplementary file 2 — Additional file 2: Chemistry data. DOC Dissolved organic carbon, DN Dissolved nitrogen, DSi Dissolved silica, TP Total phosphorus, DP Dissolved phosphorus, DRP Dissolved reactive phosphorus, A254-400 absorbance measured at corresponding wavelength (nm). [file 40793_2024_574_MOESM2_ESM.pdf]

Additional file 2: Chemistry data. DOC - dissolved organic carbon, DN - dissolved nitrogen, DSi - dissolved silica, TP - total phosphorus, DP - dissolved phosphorus, DRP dissolved reactive phosphorus, A254-400 absorbance measured at corresponding wavelength (nm).

Epilimnion 0.5m

Date	pH	DOC mg/L	DN mg/L	DSi mg/L	TP mg/L	DP µg/l	DRP µg/l	NH_4-N µg/l	NO_3-N mg/L	A254	A300	A350	A400
31-Mar-16	7.07	4.69	1.35	6.7	33.0	20.4	4.8	8.3	1.21	0.160	0.087	0.038	0.016
07-Apr-16	7.44	4.79	1.41	5.7	25.6	10.3	2.1	4.3	1.08	0.156	0.085	0.037	0.015
11-Apr-16	7.32	4.94	-	5.7	20.1	11.2	2.9	31.8	1.11	0.155	0.083	0.036	0.015
13-Apr-16	7.38	4.64	1.51	5.6	22.8	10.5	3.0	28.0	1.07	0.145	0.073	0.030	0.009
15-Apr-16	7.38	4.98	1.6	5.6	20.6	10.1	3.1	32.2	1.11	0.159	0.086	0.039	0.017
18-Apr-16	7.29	5.07	1.54	5.9	20.5	12.5	2.8	26.8	1.16	0.154	0.082	0.035	0.014
20-Apr-16	7.45	4.71	1.32	5.2	14.6	9.0	2.8	19.4	1.09	0.152	0.081	0.035	0.014
22-Apr-16	7.65	4.71	1.43	5.4	15.4	10.6	3.9	24.0	1.08	0.153	0.082	0.035	0.015
25-Apr-16	7.46	4.81	1.43	5.3	14.6	9.1	2.0	13.0	1.10	0.151	0.081	0.034	0.014
27-Apr-16	7.45	4.62	1.44	5.2	17.3	9.0	6.0	16.0	1.09	0.148	0.079	0.034	0.014
29-Apr-16	7.57	4.68	1.44	2.6	17.8	9.5	3.8	28.0	1.08	0.149	0.079	0.034	0.014
02-May-16	7.35	5.32	1.74	5.3	17.1	10.9	3.5	38.0	1.07	0.149	0.080	0.033	0.013
05-May-16	7.11	4.51	1.56	5.3	15.8	8.1	1.6	13.0	1.03	0.145	0.077	0.033	0.013
09-May-16	7.69	4.53	1.54	5.5	16.4	8.9	1.7	16.0	0.99	0.143	0.075	0.031	0.013
12-May-16	7.52	4.42	1.51	5.2	14.4	9.1	3.3	3.0	0.91	0.138	0.074	0.031	0.012
18-May-16	7.72	4.40	1.39	4.7	17.9	9.1	1.3	32.7	0.93	0.139	0.072	0.030	0.012

Hypolimnion 30m

Date	pH	DOC mg/L	DN mg/L	DSi mg/L	TP mg/L	DP µg/l	DRP µg/l	NH_4-N µg/l	NO_3-N mg/L	A254	A300	A350	A400
31-Mar-16	7.08	4.75	1.31	6.51	29.0	19.5	9.8	17.2	1.14	0.162	0.088	0.039	0.016
07-Apr-16	7.09	4.75	1.37	5.77	26.3	18.0	11.6	2.4	1.06	0.161	0.087	0.038	0.015
11-Apr-16	7.14	4.73	-	5.79	26.6	17.3	9.0	14.0	1.11	0.159	0.086	0.038	0.015
13-Apr-16	7.13	4.71	1.49	5.77	23.5	15.4	9.3	17.1	1.08	0.148	0.075	0.031	0.010
15-Apr-16	7.34	4.96	1.58	6.09	23.4	16.5	9.2	14.6	1.09	0.161	0.088	0.040	0.017
18-Apr-16	7.12	5.07	1.51	5.85	25.1	19.0	12.0	7.9	1.17	0.160	0.087	0.038	0.015
20-Apr-16	7.11	4.71	1.31	5.61	23.7	16.9	12.1	8.1	1.07	0.159	0.086	0.038	0.015
22-Apr-16	7.31	4.78	1.44	5.79	20.5	15.1	10.3	6.0	1.14	0.158	0.085	0.037	0.015
25-Apr-16	7.38	4.75	1.43	5.64	20.9	14.8	9.7	14.0	1.12	0.157	0.085	0.037	0.015
27-Apr-16	7.20	4.83	1.46	5.58	23.4	18.3	14.6	15.0	1.12	0.158	0.085	0.037	0.015
29-Apr-16	7.27	4.76	1.46	3.32	23.7	17.4	12.5	15.0	1.10	0.158	0.086	0.037	0.015
02-May-16	7.02	4.69	1.57	5.78	24.3	17.9	12.6	17.0	1.12	0.158	0.085	0.037	0.015
05-May-16	7.03	4.72	1.55	5.31	22.8	17.0	11.5	9.0	1.09	0.157	0.085	0.037	0.015
09-May-16	7.14	4.59	1.55	6.22	24.6	17.4	11.6	2.0	1.09	0.157	0.085	0.037	0.015
12-May-16	7.05	4.54	1.59	5.85	23.4	16.9	12.7	0.0	1.10	0.156	0.086	0.037	0.015
18-May-16	7.16	4.59	1.49	5.65	24.4	18.9	10.8	14.4	1.06	0.155	0.084	0.036	0.014
25-May-16	6.99	4.51	-	5.76	23.5	16.2	12.1	18.0	1.07	0.152	0.084	0.036	0.014
