# Supplementary material for: Integrating depth-dependent protist dynamics and microbial interactions in spring succession of a freshwater reservoir
Source: Environ Microbiome. 2024 May 8;19:31. doi: 10.1186/s40793-024-00574-5 (PMC11080224; doi:10.1186/s40793-024-00574-5)
Supplement: Supplementary file 3 — Additional file 3: Randomized axelerated maximum likelihood (RAxML) tree (100 bootstraps) of katablepharids. Branches with bootstrap support < 20% were multifurcated, probe targets are marked by different colors. Asterisks indicate sequences not targeted by probes. [file 40793_2024_574_MOESM3_ESM.pdf]

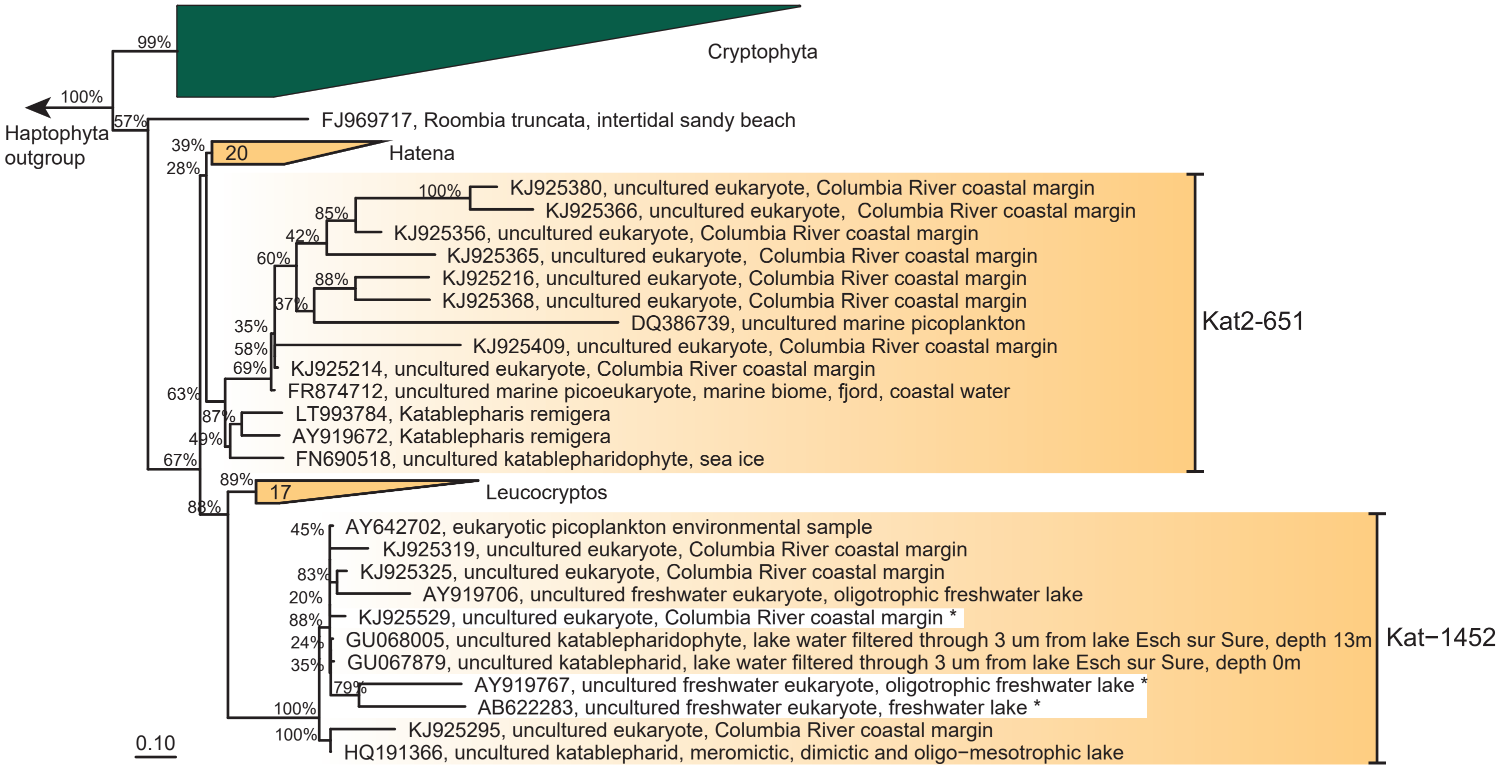

Additional file 3: Randomized axelerated maximum likelihood (RAxML) tree (100 bootstraps) of katablepharids. Branches with bootstrap support <20% were multifurcated, probe targets are marked by different colors. Asterisks indicate sequences not targeted by probes.
