# Supplementary material for: Integrating depth-dependent protist dynamics and microbial interactions in spring succession of a freshwater reservoir
Source: Environ Microbiome. 2024 May 8;19:31. doi: 10.1186/s40793-024-00574-5 (PMC11080224; doi:10.1186/s40793-024-00574-5)
Supplement: Supplementary file 6 — Additional file 6: Microphotographs displaying: a different lineages of protists hybridized with CARD-FISH probes designed for this study (Kat2-651, Telo-1250 and NC10-1290), cell hybridized with Kat-1452 shown for comparison; b different lifestyles observed for Perkinsozoa hybridized with Perkin01 (upper row free-living, lower row protist-associated). The scale bar applies for all images. Microphotographs were produced using Zeiss Imager Z2, Carl Zeiss, Oberkochen, DE equipped with a Colibri LED system and the following filter sets: DAPI 49 (Excitation 365; Beamsplitter TFT 395; Emission BP 445/50), fluorescein 38 HE (Excitation BP 470/40; Beamsplitter TFT 495; Emission BP 525/50). [file 40793_2024_574_MOESM6_ESM.pdf]

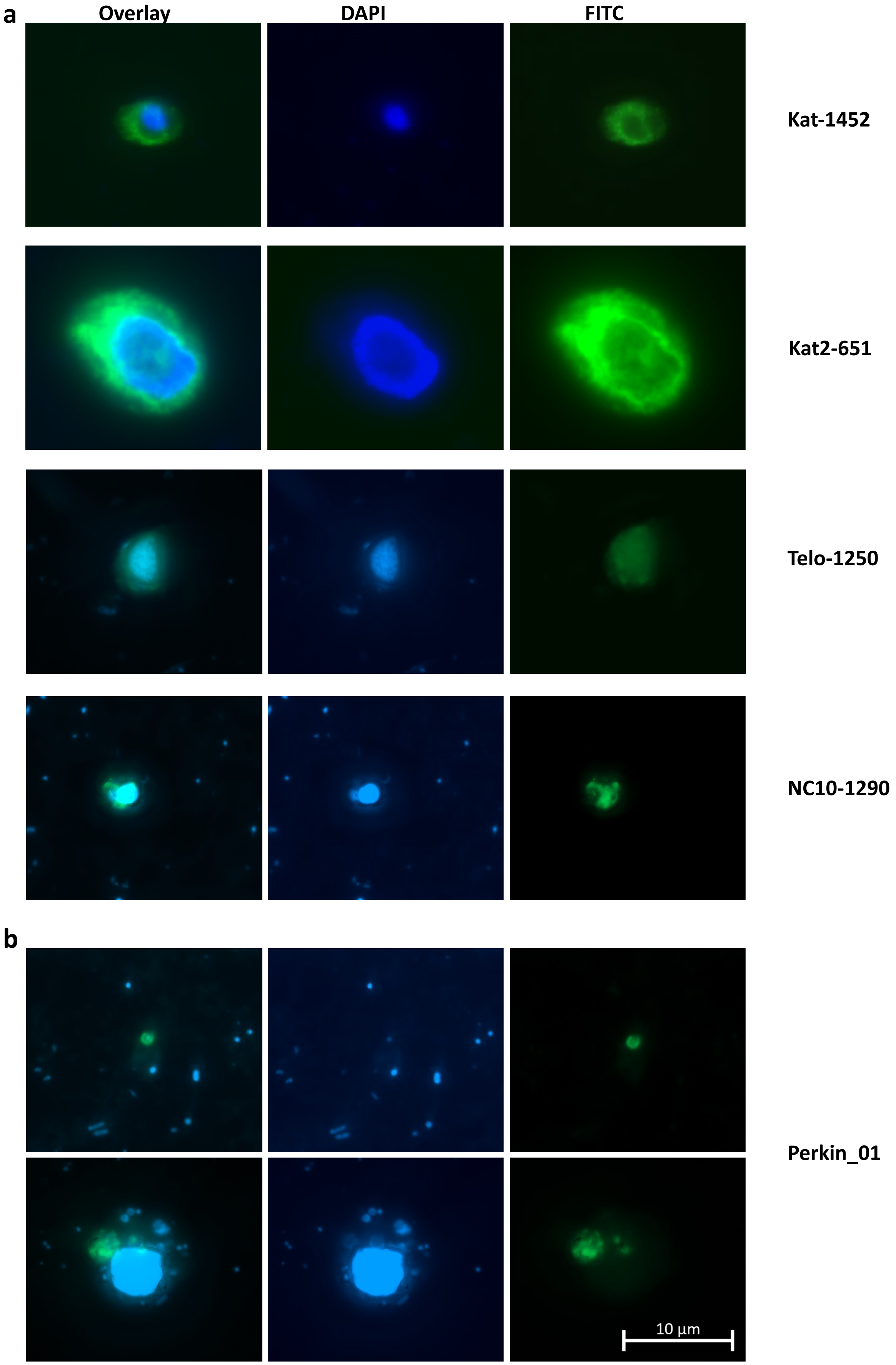

Additional file 6: Microphotographs displaying: **a.** different lineages of protists hybridized with CARD-FISH probes designed for this study (Kat2-651, Telo-1250 and NC10-1290), cell hybridized with Kat-1452 shown for comparison; **b.** different lifestyles observed for Perkinsozoa hybridized with Perkin_01 (upper row free-living, lower row protist-associated). The scale bar applies for all images.
Microphotographs were produced using Zeiss Imager Z2, Carl Zeiss, Oberkochen, DE equipped with a Colibri LED system and the following filter sets: DAPI 49 (Excitation 365; Beamsplitter TFT 395; Emission BP 445/50), fluorescein 38 HE (Excitation BP 470/40; Beamsplitter TFT 495; Emission BP 525/50).
