# Supplementary figures and images for: Integrating depth-dependent protist dynamics and microbial interactions in spring succession of a freshwater reservoir
Source: Environ Microbiome. 2024 May 8;19:31. doi: 10.1186/s40793-024-00574-5 (PMC11080224; doi:10.1186/s40793-024-00574-5)

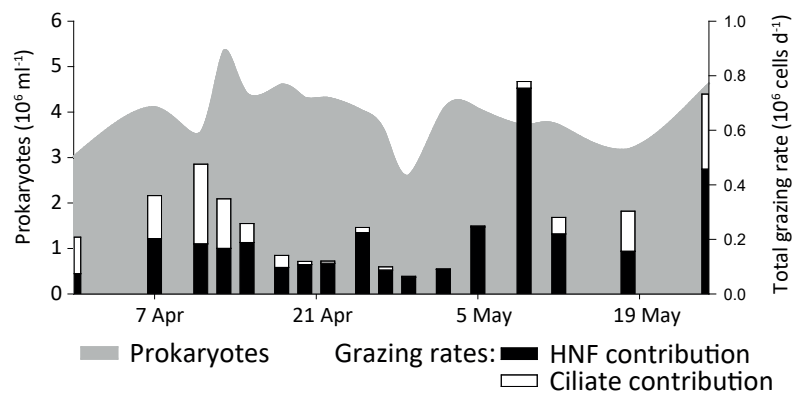

**Additional file 7:** Total grazing by protists in the epilimnion

Supplement: Supplementary file 7 — Additional file 7: Total grazing by protists in the epilimnion. [file 40793_2024_574_MOESM7_ESM.pdf]

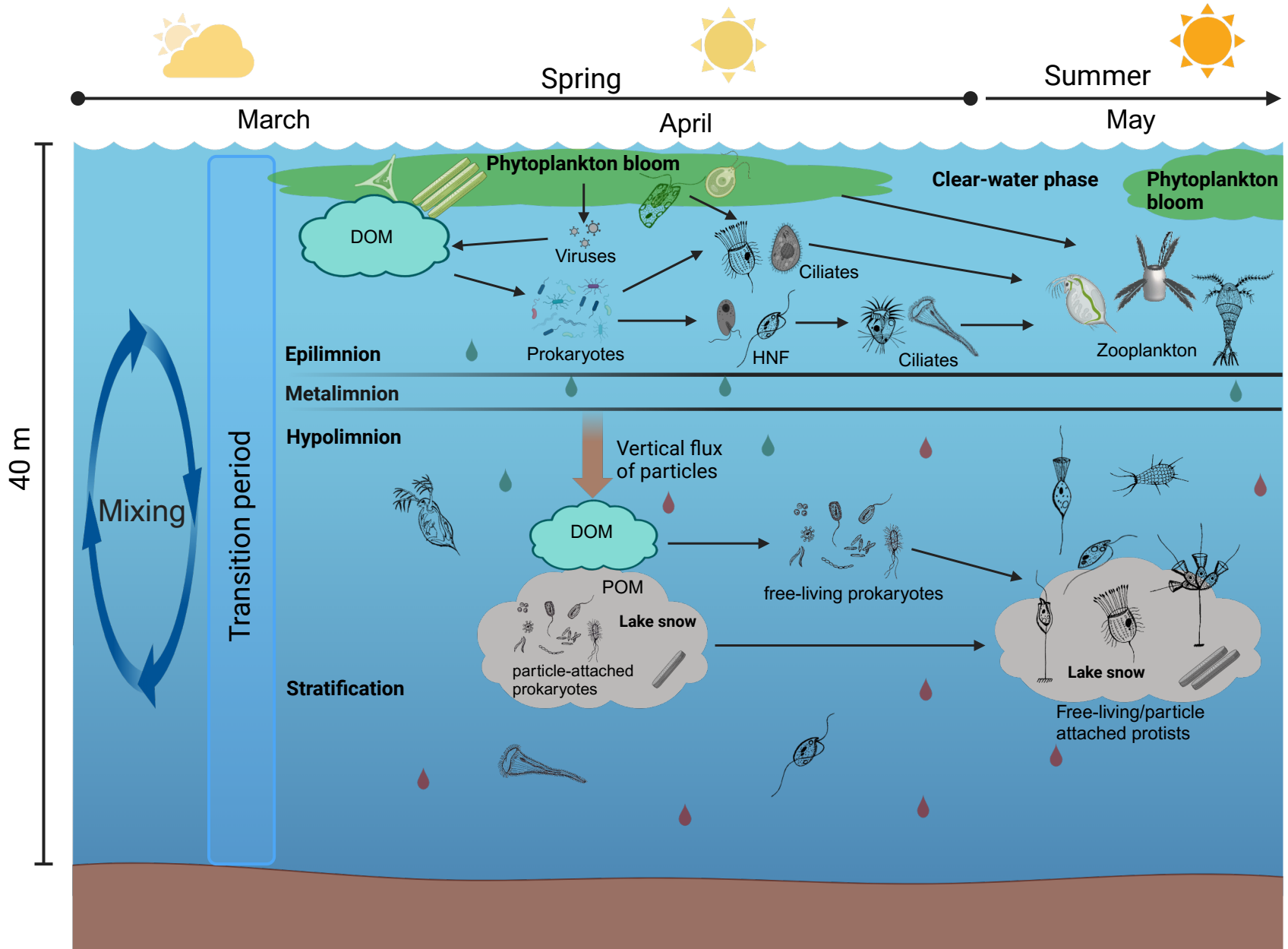

Supplement: Supplementary file 12 — Additional file 12: Schematic figure of food web in Římov reservoir during the studied spring period. Arrows indicate the direction of carbon flow. DOM Dissolved organic matter, POM Particulate organic matter, HNF Heterotrophic nanoflagellates. [file 40793_2024_574_MOESM12_ESM.pdf]
