# Supplementary material for: Integrating depth-dependent protist dynamics and microbial interactions in spring succession of a freshwater reservoir
Source: Environ Microbiome. 2024 May 8;19:31. doi: 10.1186/s40793-024-00574-5 (PMC11080224; doi:10.1186/s40793-024-00574-5)
Supplement: Supplementary file 9 — Additional file 9: Protistan community composition at three depths of Římov reservoir during the study. The gaps indicate missing samples. The groups are resolved at phylum to class level, with the exception of Supergroup Excavata, which was dominated by kinetoplastea. [file 40793_2024_574_MOESM9_ESM.pdf]

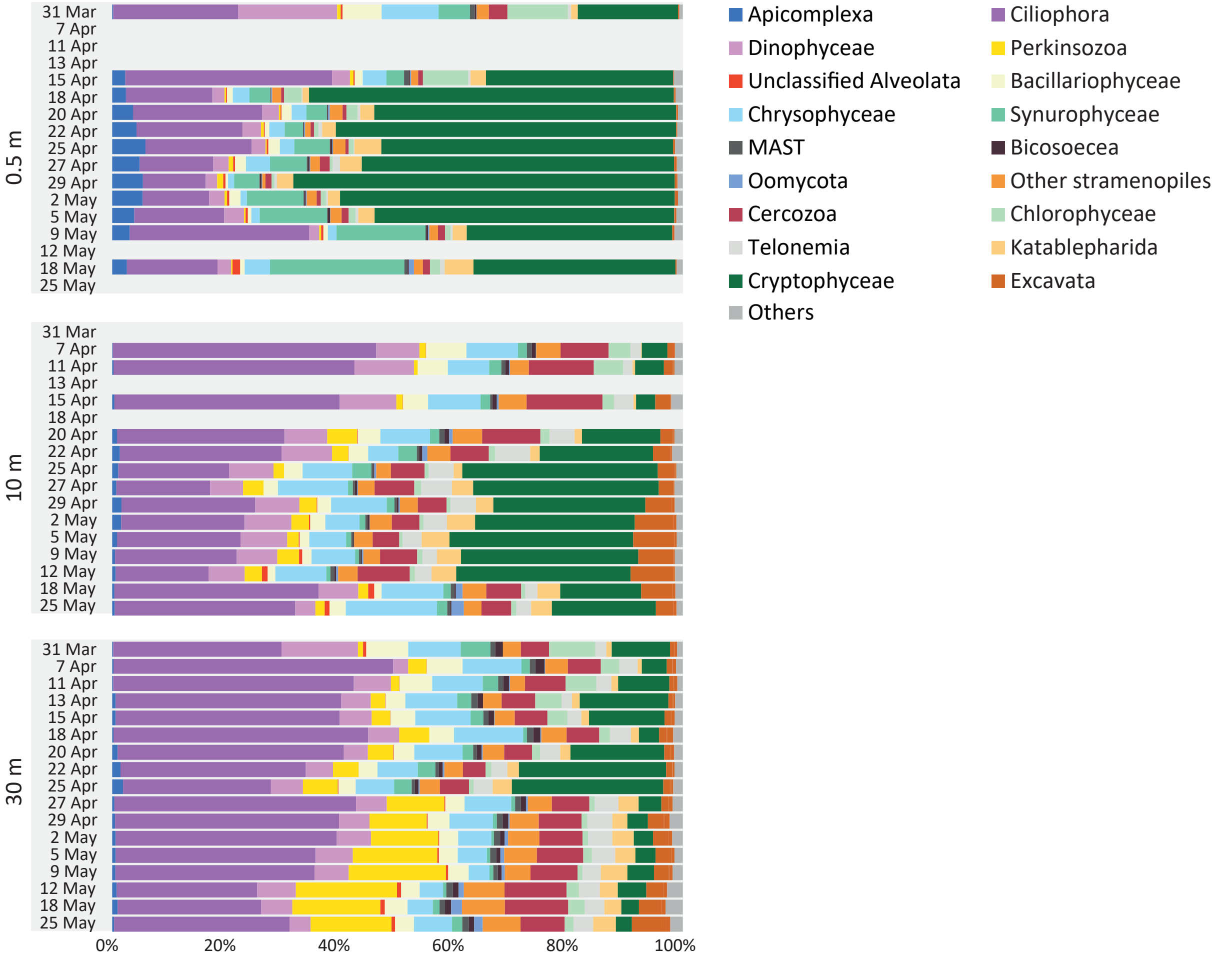

Additional file 9: Protistan community composition at three depths of Římov reservoir during the study. The gaps indicate missing samples. The groups are resolved at phylum to class level, with the exception of Supergroup Excavata, which was dominated by kinetoplastea.
