# Supplementary material for: Integrating depth-dependent protist dynamics and microbial interactions in spring succession of a freshwater reservoir
Source: Environ Microbiome. 2024 May 8;19:31. doi: 10.1186/s40793-024-00574-5 (PMC11080224; doi:10.1186/s40793-024-00574-5)
Supplement: Supplementary file 10 — Additional file 10: Relative abundances of particular flagellate groups in three depths of Římov reservoir obtained with CARD-FISH analysis. Left to right from top- cryptophytes targeted with Crypto B probe, CRY1 lineage of cryptophytes targeted with Cry1-652 probe, katablepharids targeted with Kat-1452 probe, katablepharid clade 2 targeted with Kat2-651 probe, kinetoplastids targeted with Kin516 probe, Perkinsozoa clade 1 targeted with Perkin01 probe, Telonemids targeted with Telo-1250 probe, and Cercozoa novel clade 10 targeted with NC10-1290 probe. [file 40793_2024_574_MOESM10_ESM.pdf]

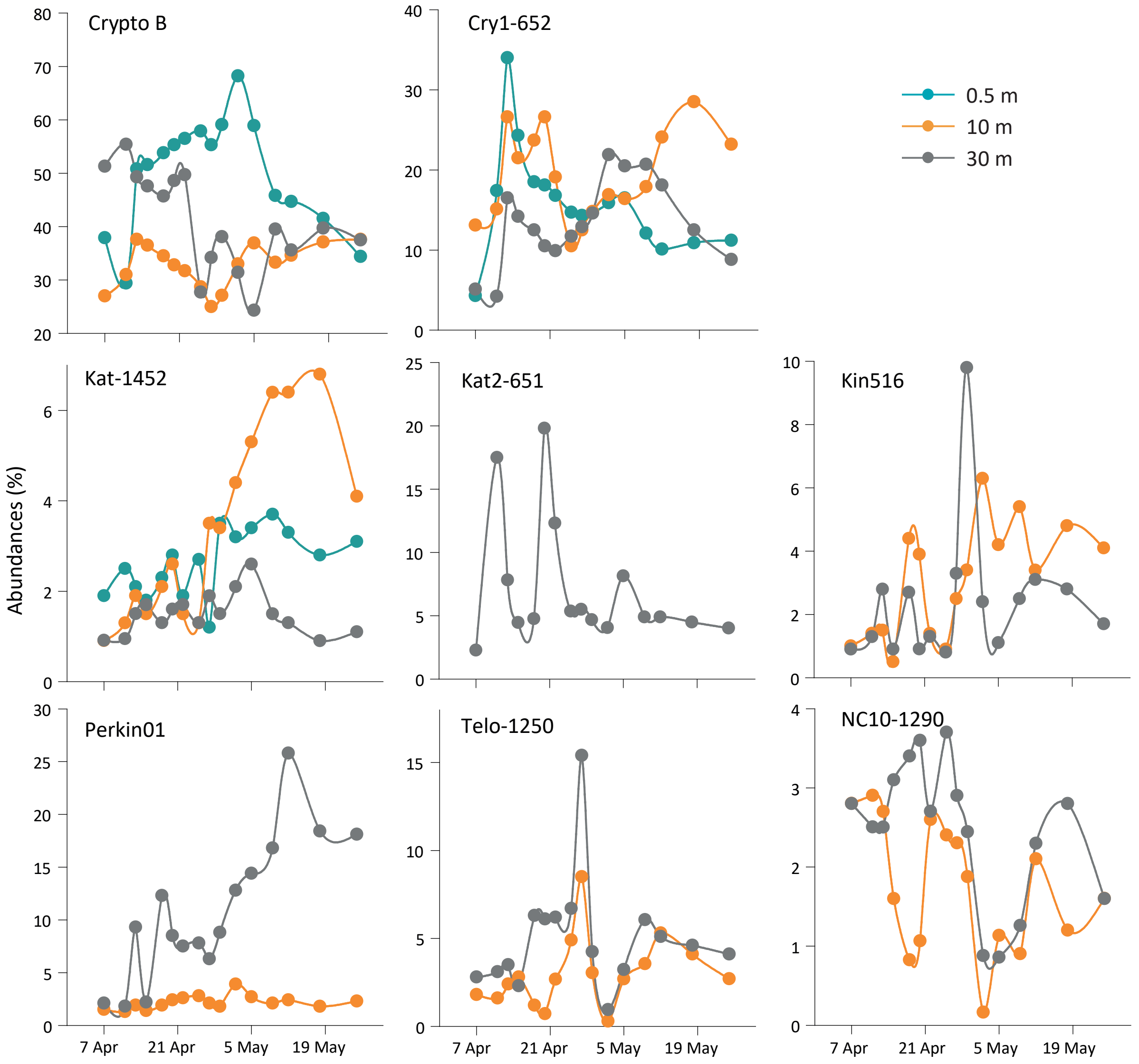

Additional file 10 : Relative abundances of particular flagellate groups in three depths of Římov reservoir obtained with CARD-FISH analysis. Left to right from top- cryptophytes targeted with Crypto B probe, CRY1 lineage of cryptophytes targeted with Cry1-652 probe, katablepharids targeted with Kat-1452 probe, katablepharid clade 2 targeted with Kat2-651 probe, kinetoplastids targeted with Kin516 probe, Perkinsozoa clade 1 targeted with Perkin01 probe, Telonemids targeted with Telo-1250 probe, and Cercozoa novel clade 10 targeted with NC10-1290 probe.
