# Supplementary material for: Integrating depth-dependent protist dynamics and microbial interactions in spring succession of a freshwater reservoir
Source: Environ Microbiome. 2024 May 8;19:31. doi: 10.1186/s40793-024-00574-5 (PMC11080224; doi:10.1186/s40793-024-00574-5)

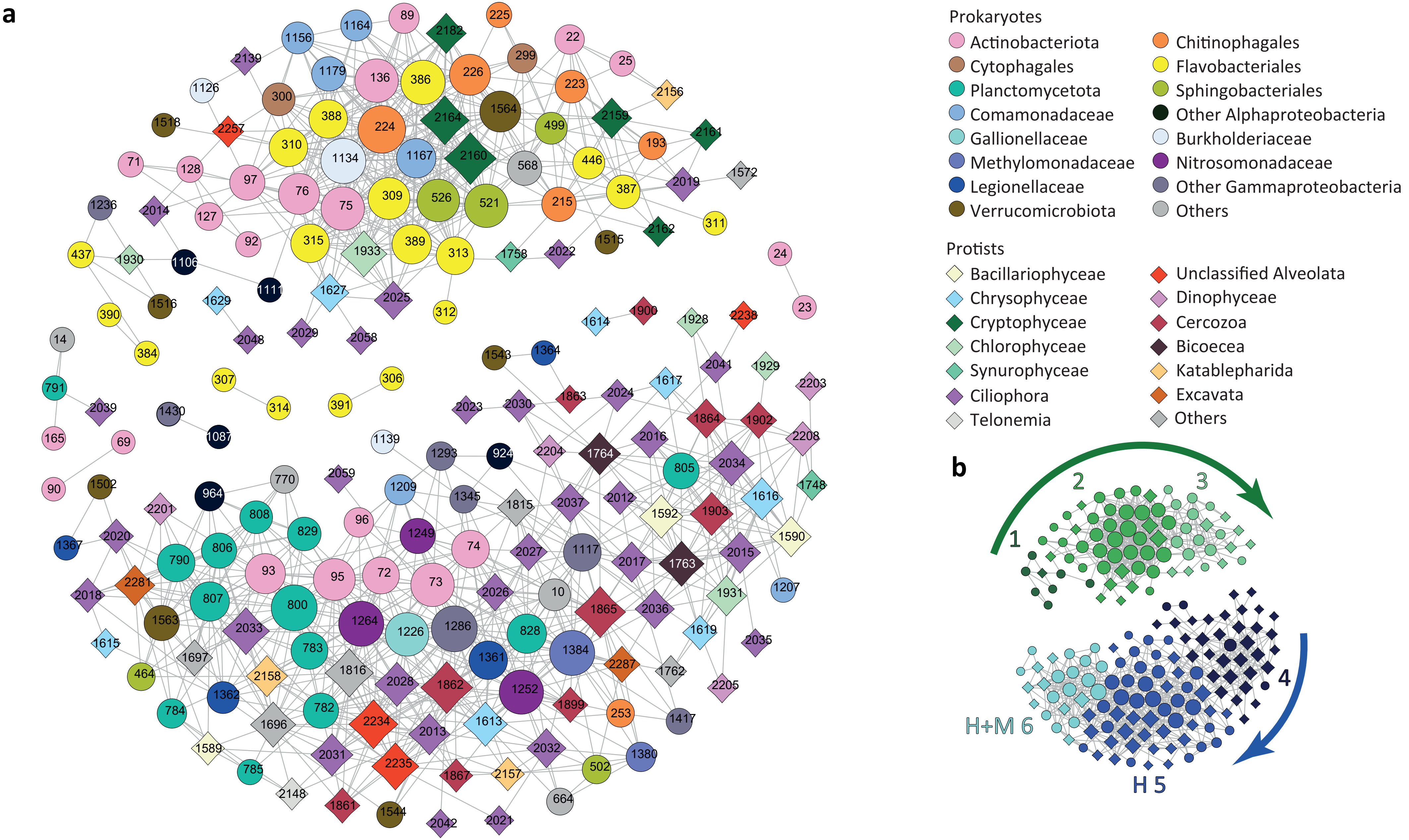

Additional file 11:

Network analysis based on the most abundant ASVs from protistan and prokaryotic communities. **a** – network: upper cluster represents the community dominating in the epilimnion, lower cluster represents the community dominating in the hypolimnion. Prokaryotic nodes are displayed as circles, protistan nodes as diamonds. **b** – the main modules detected in the network. The arrows indicate directions of temporal shifts between modules. Differentiation between modules H 5 and H+M 6 is based on spatial parameters as members of module 6 were better resented in the metalimnion communities. Prokaryotic and protistan nodes are organized into modules and listed below, accompanied by heatmaps based on Z scores calculated for each module. Samples are grouped according to water column layers, with the time course depicted from left to right.

Module 1

Phylum	Class	Order	Family	Genus	ID
Bacteroidota	Bacteroidia	Flavobacteriales	Flavobacteriaceae	Flavobacterium	384
Bacteroidota	Bacteroidia	Flavobacteriales	Flavobacteriaceae	Flavobacterium	390
Bacteroidota	Bacteroidia	Flavobacteriales	Flavobacteriaceae	Flavobacterium	437
Verrucomicrobiota	Verrucomicrobiae	NA	NA	NA	1516
Proteobacteria	Gammaproteobacteria	Burkholderiales	Methylophilaceae	Methylotenera	1236
Proteobacteria	Alphaproteobacteria	Sphingomonadales	Sphingomonadaceae	Sphingorhabdus	1106
Proteobacteria	Alphaproteobacteria	Sphingomonadales	Sphingomonadaceae	Sphingorhabdus	1111
Archaeplastida	Chlorophyta	Chlorophyceae	Chlamydomonadales	Chlamydomonadales_X	1930

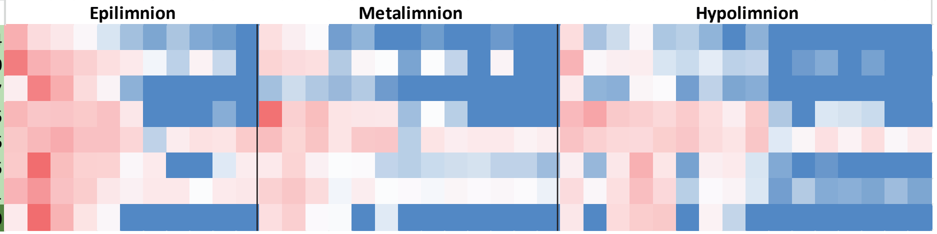

Module 2

Phylum	Class	Order	Family	Genus	ID
Actinobacteriota	Actinobacteria	Frankiales	Sporichthyaceae	Candidatus Planktophila	71
Actinobacteriota	Actinobacteria	Frankiales	Sporichthyaceae	Candidatus Planktophila	75
Actinobacteriota	Actinobacteria	Frankiales	Sporichthyaceae	Candidatus Planktophila	76
Actinobacteriota	Actinobacteria	Frankiales	Sporichthyaceae	hgcI clade	89
Actinobacteriota	Actinobacteria	Frankiales	Sporichthyaceae	hgcI clade	92
Actinobacteriota	Actinobacteria	Frankiales	Sporichthyaceae	hgcI clade	97
Actinobacteriota	Actinobacteria	Frankiales	Sporichthyaceae	NA	127
Actinobacteriota	Actinobacteria	Frankiales	Sporichthyaceae	NA	128
Actinobacteriota	Actinobacteria	Micrococcales	Microbacteriaceae	Candidatus Limnoluna	136
Bacteroidota	Bacteroidia	Chitinophagales	Chitinophagaceae	Sediminibacterium	224
Bacteroidota	Bacteroidia	Chitinophagales	Chitinophagaceae	Sediminibacterium	225
Bacteroidota	Bacteroidia	Chitinophagales	Chitinophagaceae	Sediminibacterium	226
Bacteroidota	Bacteroidia	Cytophagales	Spirosomaceae	Pseudarcicella	299
Bacteroidota	Bacteroidia	Cytophagales	Spirosomaceae	Pseudarcicella	300
Bacteroidota	Bacteroidia	Flavobacteriales	Crocinitomicaceae	Fluviicola	309
Bacteroidota	Bacteroidia	Flavobacteriales	Crocinitomicaceae	Fluviicola	310
Bacteroidota	Bacteroidia	Flavobacteriales	Crocinitomicaceae	Fluviicola	312
Bacteroidota	Bacteroidia	Flavobacteriales	Crocinitomicaceae	Fluviicola	313
Bacteroidota	Bacteroidia	Flavobacteriales	Crocinitomicaceae	Fluviicola	315
Bacteroidota	Bacteroidia	Flavobacteriales	Flavobacteriaceae	Flavobacterium	386
Bacteroidota	Bacteroidia	Flavobacteriales	Flavobacteriaceae	Flavobacterium	387
Bacteroidota	Bacteroidia	Flavobacteriales	Flavobacteriaceae	Flavobacterium	388
Bacteroidota	Bacteroidia	Flavobacteriales	Flavobacteriaceae	Flavobacterium	389
Bacteroidota	Bacteroidia	Sphingobacteriales	Sphingobacteriaceae	Pedobacter	521
Bacteroidota	Bacteroidia	Sphingobacteriales	Sphingobacteriaceae	Solitalea	526
Proteobacteria	Gammaproteobacteria	Burkholderiales	Burkholderiaceae	Polynucleobacter	1126
Proteobacteria	Gammaproteobacteria	Burkholderiales	Burkholderiaceae	Polynucleobacter	1134
Proteobacteria	Gammaproteobacteria	Burkholderiales	Comamonadaceae	Acidovorax	1156
Proteobacteria	Gammaproteobacteria	Burkholderiales	Comamonadaceae	Limnohabitans	1164
Proteobacteria	Gammaproteobacteria	Burkholderiales	Comamonadaceae	Limnohabitans	1167
Proteobacteria	Gammaproteobacteria	Burkholderiales	Comamonadaceae	NA	1179
Verrucomicrobiota	Verrucomicrobiae	NA	NA	NA	1518
Verrucomicrobiota	Verrucomicrobiae	Verrucomicrobiales	Verrucomicrobiaceae	NA	1564
Alveolata	Ciliophora	Spirotrichea	Strombidiida	Pelagostrombidiidae	2014
Hacrobia	Haptophyta	Prymnesiophyceae	Prymnesiales	Chrysochromulinaceae	2257
Alveolata	Ciliophora	NA	NA	NA	2139
Stramenopiles	Ochrophyta	Chrysophyceae	Chrysophyceae_X	NA	1627
Alveolata	Ciliophora	Oligohymenophorea	Peritrichia_2	Sessilida	2058
Alveolata	Ciliophora	Spirotrichea	Tintinnida	TIN_03	2029
Alveolata	Ciliophora	Spirotrichea	Hypotrichia	Oxytrichidae	2025
Hacrobia	Cryptophyta	Cryptophyceae	Cryptomonadales	Cryptomonadales_X	2164
Hacrobia	Cryptophyta	NA	NA	NA	2182
Archaeplastida	Chlorophyta	Chlorophyceae	Chlamydomonadales	Chlamydomonadales_X	1933
Hacrobia	Cryptophyta	Cryptophyceae	Cryptomonadales	Cryptomonadales_X	2160

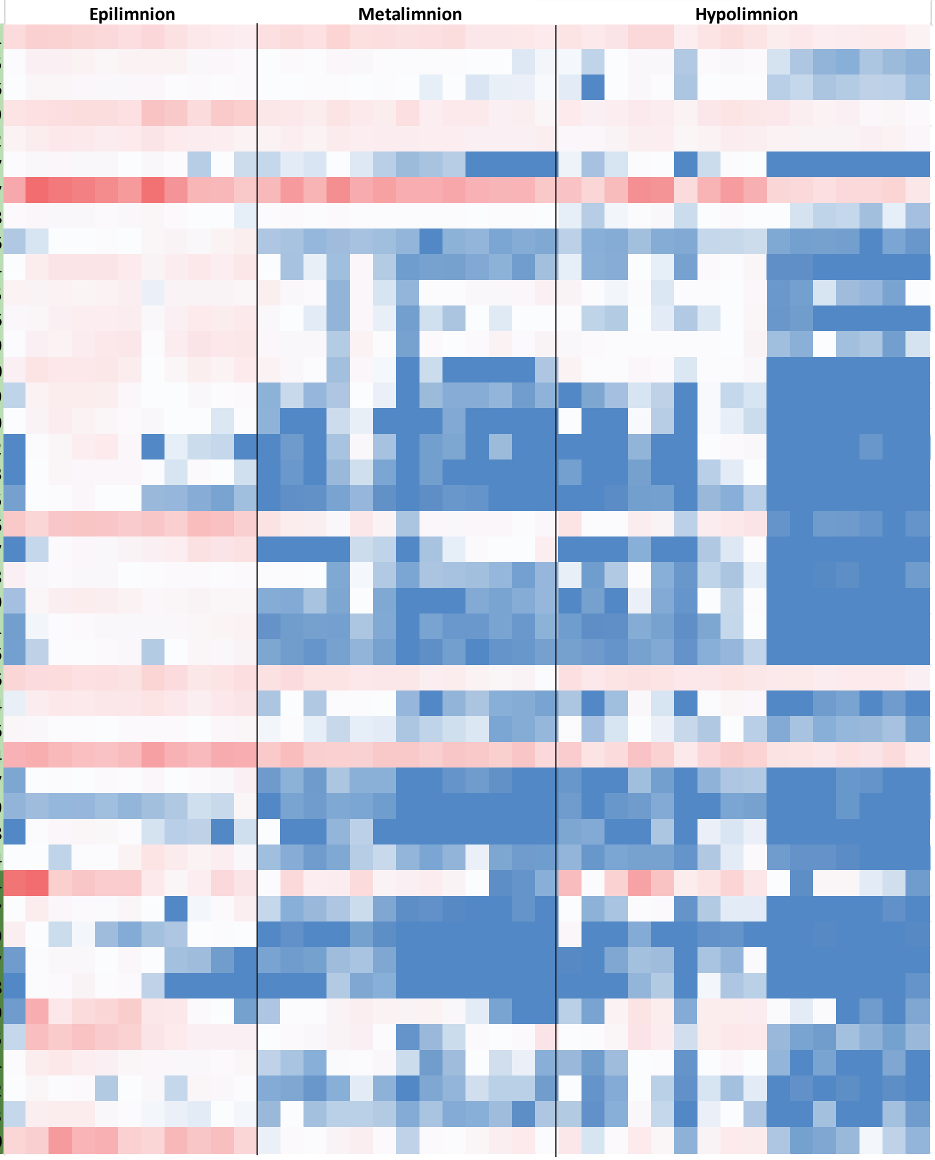

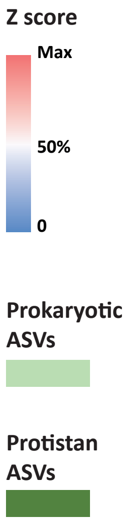

Module 3

Phylum	Class	Order	Family	Genus	ID
Chloroflexi	SL56 marine group	NA	NA	NA	568
Bacteroidota	Bacteroidia	Chitinophagales	Chitinophagaceae	NA	215
Verrucomicrobiota	Verrucomicrobiae	NA	NA	NA	1515
Bacteroidota	Bacteroidia	Flavobacteriales	Crocinitomicaceae	Fluviicola	311
Bacteroidota	Bacteroidia	Flavobacteriales	NS9 marine group	NA	446
Bacteroidota	Bacteroidia	Chitinophagales	Chitinophagaceae	Edaphobaculum	193
Bacteroidota	Bacteroidia	Sphingobacteriales	NS11-12 marine group	NA	499
Bacteroidota	Bacteroidia	Chitinophagales	Chitinophagaceae	Sediminibacterium	223
Actinobacteriota	Acidimicrobiia	Microtrichales	Ilumatobacteraceae	CL500-29 marine group	22
Actinobacteriota	Acidimicrobiia	Microtrichales	Ilumatobacteraceae	CL500-29 marine group	25
Alveolata	Apicomplexa	Coccidiomorphea	Adeleida	NA	1572
Alveolata	Ciliophora	Spirotrichea	Strombidiida_A	Strombidiida_A_X	2022
Hacrobia	Katablepharidophyta	Katablepharidaceae	Katablepharidales	Katablepharidales_X	2156
Hacrobia	Cryptophyta	Cryptophyceae	Cryptomonadales	Cryptomonadales_X	2159
Hacrobia	Cryptophyta	Cryptophyceae	Cryptomonadales	Cryptomonadales_X	2161
Hacrobia	Cryptophyta	Cryptophyceae	Cryptomonadales	Cryptomonadales_X	2162
Stramenopiles	Ochrophyta	Synurophyceae	Synurales	Synurales_X	1758
Alveolata	Ciliophora	Spirotrichea	Choreotrichida	Strobilidiidae_D	2019

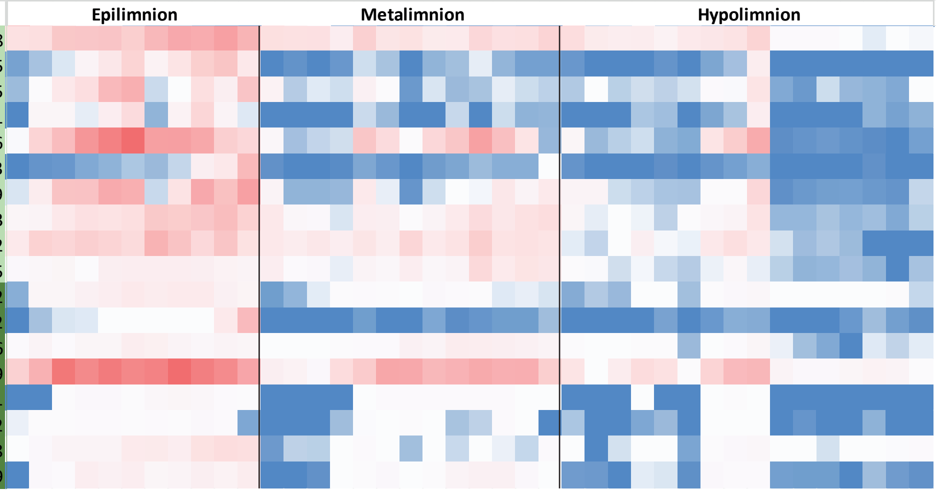

Module 4

Phylum	Class	Order	Family	Genus	ID
Planctomycetota	Planctomycetes	Pirellulales	Pirellulaceae	NA	805
Verrucomicrobiota	Verrucomicrobiae	Pedosphaerales	Pedosphaeraceae	NA	1543
Proteobacteria	Gammaproteobacteria	Legionellales	Legionellaceae	Legionella	1364
Proteobacteria	Gammaproteobacteria	Burkholderiales	Comamonadaceae	Polaromonas	1207
Stramenopiles	Ochrophyta	Bacillariophyta	Bacillariophyta_X	Polar-centric-Mediophyceae	1590
Stramenopiles	Ochrophyta	Bacillariophyta	Bacillariophyta_X	Polar-centric-Mediophyceae	1592
Stramenopiles	Ochrophyta	Chrysophyceae	Chrysophyceae_X	Chrysophyceae_Clade-C	1616
Stramenopiles	Ochrophyta	Chrysophyceae	Chrysophyceae_X	NA	1617
Stramenopiles	Ochrophyta	Chrysophyceae	Chrysophyceae_X	Chrysophyceae_Clade-E	1619
Stramenopiles	Ochrophyta	Synurophyceae	Synurales	Synurales_X	1748
Stramenopiles	Opalozoa	Bicoecea	Bicoecales	Bicoecaceae	1763
Stramenopiles	Opalozoa	Bicoecea	Bicoecales	Bicoecaceae	1764
Rhizaria	Cercozoa	NA	NA	NA	1863
Rhizaria	Cercozoa	Filosa-Thecofilosea	Ebriida	Botuliformidae	1864
Rhizaria	Cercozoa	NA	NA	NA	1902
Rhizaria	Cercozoa	NA	NA	NA	1903
Archaeplastida	Chlorophyta	Chlorophyceae	Chlamydomonadales	Chlamydomonadales_X	1928
Archaeplastida	Chlorophyta	Chlorophyceae	Chlamydomonadales	Chlamydomonadales_X	1929
Archaeplastida	Chlorophyta	Chlorophyceae	Chlamydomonadales	Chlamydomonadales_X	1931
Alveolata	Ciliophora	Oligohymenophorea	Scuticociliatia_2	Histiobalantiidae	2012
Alveolata	Ciliophora	Spirotrichea	Tintinnida	Tintinnidiidae	2015
Alveolata	Ciliophora	Oligohymenophorea	Peritrichia_2	Sessilida	2016
Alveolata	Ciliophora	Spirotrichea	Hypotrichia	NA	2017
Alveolata	Ciliophora	Litostomatea	Litostomatea_X	Litostomatea_XX	2023
Alveolata	Ciliophora	Colpodea	Colpodea_X	NA	2024
Alveolata	Ciliophora	NA	NA	NA	2030
Alveolata	Ciliophora	Oligohymenophorea	Peritrichia_2	Sessilida	2034
Alveolata	Ciliophora	Spirotrichea	Tintinnida	Tintinnidiidae	2035
Alveolata	Ciliophora	Litostomatea	NA	NA	2036
Alveolata	Ciliophora	Phyllopharyngea	Suctoria	Tokophryidae	2037
Alveolata	Ciliophora	Oligohymenophorea	Peritrichia_2	Sessilida	2041
Alveolata	Dinoflagellata	Dinophyceae	Gymnodiniales	Gymnodiniaceae	2203
Alveolata	Dinoflagellata	Dinophyceae	Prorocentrales	Prorocentraceae	2204
Alveolata	Dinoflagellata	Dinophyceae	Prorocentrales	Prorocentraceae	2205
Alveolata	Dinoflagellata	Dinophyceae	NA	NA	2208
Alveolata	NA	NA	NA	NA	2238

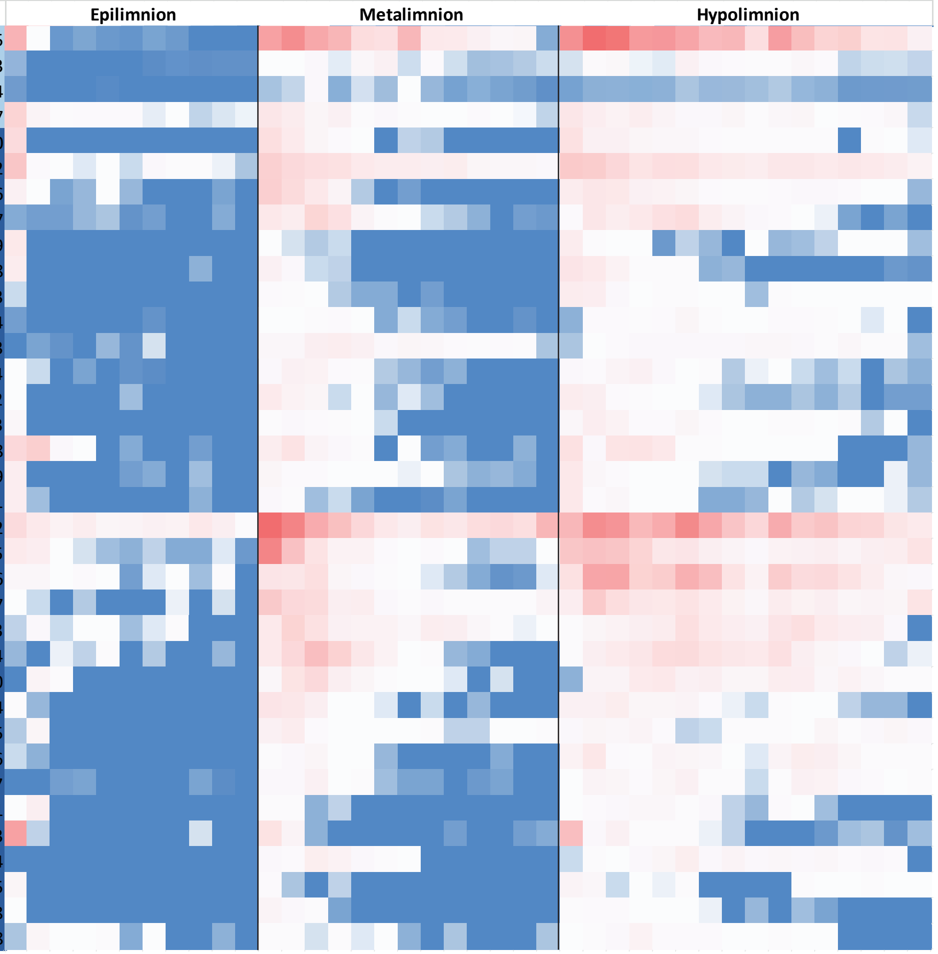

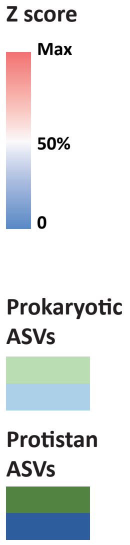

Module 5

Phylum	Class	Order	Family	Genus	ID
Acidobacteriota	Vicinamibacteria	Vicinamibacterales	NA	NA	10
Actinobacteriota	Actinobacteria	Frankiales	Sporichthyaceae	Candidatus Planktophila	72
Actinobacteriota	Actinobacteria	Frankiales	Sporichthyaceae	Candidatus Planktophila	73
Actinobacteriota	Actinobacteria	Frankiales	Sporichthyaceae	Candidatus Planktophila	74
Actinobacteriota	Actinobacteria	Frankiales	Sporichthyaceae	hgcI clade	95
Actinobacteriota	Actinobacteria	Frankiales	Sporichthyaceae	hgcI clade	96
Bacteroidota	Bacteroidia	Chitinophagales	NA	NA	253
Bacteroidota	Bacteroidia	Sphingobacteriales	NS11-12 marine group	NA	502
Myxococcota	Polyangia	mle1-27	NA	NA	664
Planctomycetota	Phycisphaerae	Phycisphaerales	Phycisphaeraceae	CL500-3	782
Planctomycetota	Phycisphaerae	Phycisphaerales	Phycisphaeraceae	CL500-3	783
Planctomycetota	Phycisphaerae	Phycisphaerales	Phycisphaeraceae	CL500-3	785
Planctomycetota	Planctomycetes	Pirellulales	Pirellulaceae	NA	808
Planctomycetota	Planctomycetes	Planctomycetales	Rubinisphaeraceae	SH-PL14	828
Proteobacteria	Alphaproteobacteria	Rhizobiales	Beijerinckiaceae	alphaI cluster	924
Proteobacteria	Gammaproteobacteria	Burkholderiales	Alcaligenaceae	GKS98 freshwater group	1117
Proteobacteria	Gammaproteobacteria	Burkholderiales	Burkholderiaceae	Polynucleobacter	1139
Proteobacteria	Gammaproteobacteria	Burkholderiales	Comamonadaceae	Rhizobacter	1209
Proteobacteria	Gammaproteobacteria	Burkholderiales	Gallionellaceae	Candidatus Nitrotoga	1226
Proteobacteria	Gammaproteobacteria	Burkholderiales	Nitrosomonadaceae	GOUTA6	1249
Proteobacteria	Gammaproteobacteria	Burkholderiales	Nitrosomonadaceae	NA	1252
Proteobacteria	Gammaproteobacteria	Burkholderiales	Nitrosomonadaceae	Nitrosospira	1264
Proteobacteria	Gammaproteobacteria	Burkholderiales	SC-I-84	NA	1286
Proteobacteria	Gammaproteobacteria	Burkholderiales	TRA3-20	NA	1293
Proteobacteria	Gammaproteobacteria	Diplorickettsiales	Diplorickettsiaceae	Rickettsiella	1345
Proteobacteria	Gammaproteobacteria	Legionellales	Legionellaceae	Legionella	1361
Proteobacteria	Gammaproteobacteria	Methylococcales	Methylomonadaceae	Methylobacter	1380
Proteobacteria	Gammaproteobacteria	Methylococcales	Methylomonadaceae	Methylobacter	1384
Proteobacteria	Gammaproteobacteria	Pseudomonadales	Pseudohongiellaceae	Blyi10	1417
Verrucomicrobiota	Verrucomicrobiae	Pedosphaerales	Pedosphaeraceae	NA	1544
Verrucomicrobiota	Verrucomicrobiae	Verrucomicrobiales	Verrucomicrobiaceae	NA	1563
Stramenopiles	Ochrophyta	Chrysophyceae	Chrysophyceae_X	NA	1613
Stramenopiles	Ochrophyta	NA	NA	NA	1696
Stramenopiles	Pseudofungi	MAST-2	MAST-2C	MAST-2C_X	1762
Stramenopiles	NA	NA	NA	NA	1815
Stramenopiles	NA	NA	NA	NA	1816
Rhizaria	Cercozoa	Filosa-Imbricatea	Filosa-Imbricatea_X	Novel-clade-2	1861
Rhizaria	Cercozoa	Filosa-Imbricatea	Thaumatomonadida	Peregriniidae	1862
Rhizaria	Cercozoa	Filosa-Thecofilosea	Cryomonadida	Cryothecomonas-lineage	1865
Rhizaria	Cercozoa	Filosa-Thecofilosea	Cryomonadida	NA	1867
Rhizaria	Cercozoa	NA	NA	NA	1899
Alveolata	Ciliophora	NA	NA	NA	2013
Alveolata	Ciliophora	Oligohymenophorea	Hymenostomatia	Ophryoglenida	2021
Alveolata	Ciliophora	Spirotrichea	Choreotrichida	NA	2026
Alveolata	Ciliophora	NA	NA	NA	2027
Alveolata	Ciliophora	NA	NA	NA	2028
Alveolata	Ciliophora	NA	NA	NA	2031
Alveolata	Ciliophora	NA	NA	NA	2032
Alveolata	Ciliophora	Litostomatea	NA	NA	2042
Alveolata	Ciliophora	Ciliophora_X	Ciliophora_XX	Ciliophora_XXX	2059
Hacrobia	Telonemia	Telonemia_X	Telonemia_XX	Telonemia-Group-2	2148
Hacrobia	Katablepharidophyta	Katablepharidaceae	Katablepharidales	Katablepharidales_X	2157
Hacrobia	Katablepharidophyta	Katablepharidaceae	Katablepharidales	Katablepharidales_X	2158
Alveolata	NA	NA	NA	NA	2234
Alveolata	NA	NA	NA	NA	2235
Excavata	Discoba	Kinetoplastea	Eubodonida	Bodonidae	2287

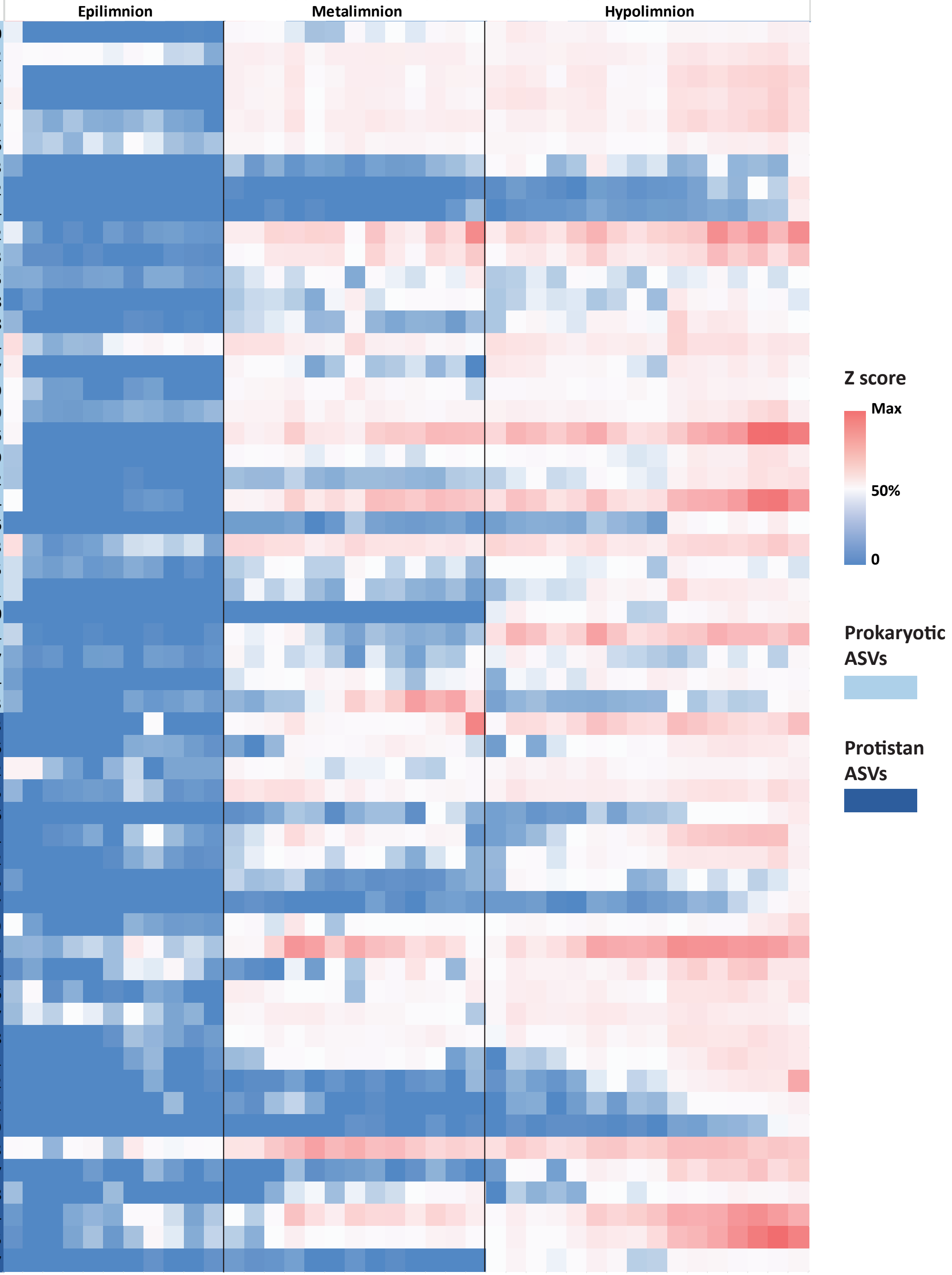

Module 6

Phylum	Class	Order	Family	Genus	ID
Planctomycetota	Planctomycetes	Gemmatales	Gemmataceae	NA	790
Planctomycetota	Planctomycetes	Isosphaerales	Isosphaeraceae	NA	800
Planctomycetota	Planctomycetes	Pirellulales	Pirellulaceae	NA	806
Planctomycetota	Planctomycetes	Pirellulales	Pirellulaceae	NA	807
Actinobacteriota	Actinobacteria	Frankiales	Sporichthyaceae	hgcI clade	93
Verrucomicrobiota	Verrucomicrobiae	Chthoniobacterales	Chthoniobacteraceae	Chthoniobacter	1502
Proteobacteria	Gammaproteobacteria	Legionellales	Legionellaceae	Legionella	1367
Bacteroidota	Bacteroidia	Sphingobacteriales	env.OPS 17	NA	464
Planctomycetota	Phycisphaerae	Phycisphaerales	Phycisphaeraceae	CL500-3	784
Proteobacteria	Alphaproteobacteria	Rhizobiales	Rhizobiales Incertae Se	NA	964
Patescibacteria	Saccharimonadia	Saccharimonadales	NA	NA	770
Planctomycetota	Planctomycetes	Planctomycetales	Schlesneriaceae	Schlesneria	829
Proteobacteria	Gammaproteobacteria	Legionellales	Legionellaceae	Legionella	1362
Stramenopiles	Ochrophyta	Bacillariophyta	Bacillariophyta_X	Araphid-pennate	1589
Stramenopiles	Ochrophyta	Chrysophyceae	Chrysophyceae_X	Chrysophyceae_Clade-E	1615
Stramenopiles	Ochrophyta	NA	NA	NA	1697
Alveolata	Ciliophora	Spirotrichea	Choreotrichida	NA	2018
Alveolata	Ciliophora	Litostomatea	Litostomatea_X	Litostomatea_XX	2020
Alveolata	Ciliophora	Spirotrichea	Choreotrichida	NA	2033
Alveolata	Dinoflagellata	Dinophyceae	Peridiniales	Thoracosphaeraceae	2201
Excavata	Discoba	NA	NA	NA	2281

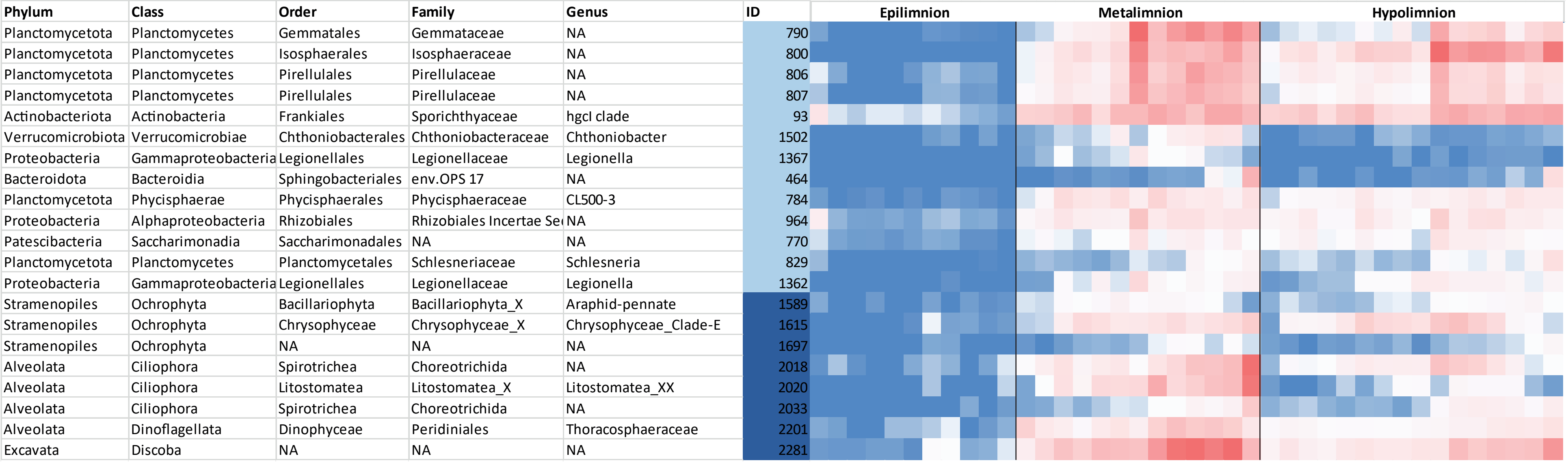

Diverse small modules

Phylum	Class	Order	Family	Genus	ID
Acidobacteriota	Vicinamibacteria	Vicinamibacterales	Vicinamibacteraceae	NA	14
Actinobacteriota	Acidimicrobiia	Microtrichales	Ilumatobacteraceae	CL500-29 marine group	23
Actinobacteriota	Acidimicrobiia	Microtrichales	Ilumatobacteraceae	CL500-29 marine group	24
Actinobacteriota	Actinobacteria	Frankiales	Sporichthyaceae	Candidatus Planktophila	69
Actinobacteriota	Actinobacteria	Frankiales	Sporichthyaceae	hgcI clade	90
Actinobacteriota	Thermoleophilia	Gaiellales	NA	NA	165
Bacteroidota	Bacteroidia	Flavobacteriales	Crocinitomicaceae	Fluviicola	306
Bacteroidota	Bacteroidia	Flavobacteriales	Crocinitomicaceae	Fluviicola	307
Bacteroidota	Bacteroidia	Flavobacteriales	Crocinitomicaceae	Fluviicola	314
Bacteroidota	Bacteroidia	Flavobacteriales	Flavobacteriaceae	Flavobacterium	391
Planctomycetota	Planctomycetes	Gemmatales	Gemmataceae	NA	791
Proteobacteria	Alphaproteobacteria	Sphingomonadales	Sphingomonadaceae	Sphingobium	1087
Proteobacteria	Gammaproteobacteria	Salinisphaerales	Solimonadaceae	NA	1430
Alveolata	Ciliophora	Litostomatea	Litostomatea_X	Litostomatea_XX	2048
Alveolata	Ciliophora	NA	NA	NA	2039
Stramenopiles	Ochrophyta	Chrysophyceae	Chrysophyceae_X	NA	1614
Stramenopiles	Ochrophyta	Chrysophyceae	Chrysophyceae_X	NA	1629
Rhizaria	Cercozoa	NA	NA	NA	1900

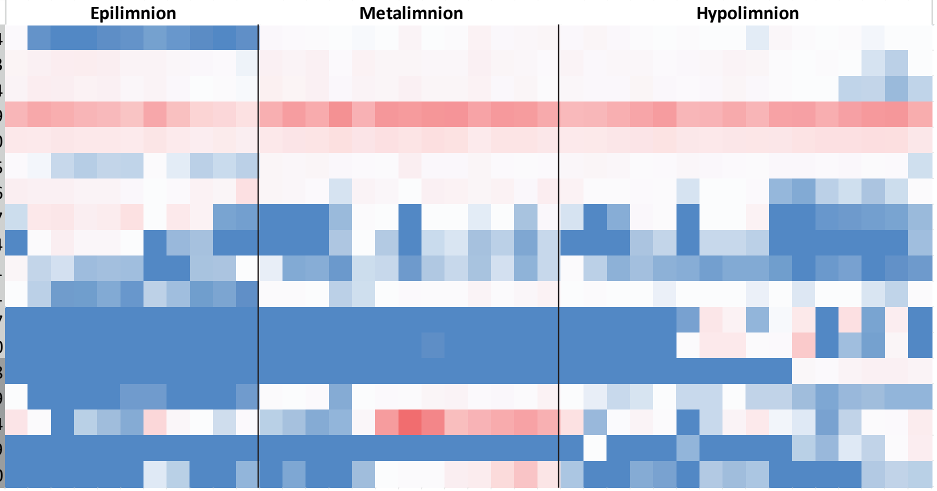

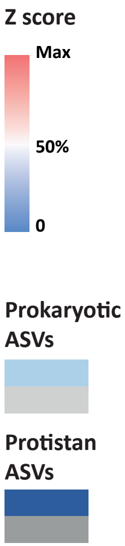

Supplement: Supplementary file 11 — Additional file 11: Network analysis based on the most abundant ASVs from protistan and prokaryotic communities. a Network: upper cluster represents the community dominating in the epilimnion, lower cluster represents the community dominating in the hypolimnion. Prokaryotic nodes are displayed as circles, protistan nodes as diamonds. b The main modules detected in the network. The arrows indicate directions of temporal shifts between modules. Differentiation between modules H 5 and H+M 6 is based on spatial parameters as members of module 6 were better resented in the metalimnion communities. Prokaryotic and protistan nodes are organized into modules and listed below, accompanied by heatmaps based on Z scores calculated for each module. Samples are grouped according to water column layers, with the time course depicted from left to right. [file 40793_2024_574_MOESM11_ESM.pdf]
